# Integration of mRNA and miRNA Analysis Reveals the Molecular Mechanism of Cotton Response to Salt Stress

**DOI:** 10.3389/fpls.2021.767984

**Published:** 2021-12-09

**Authors:** Jingjing Zhan, Yangyang Diao, Guo Yin, Muhammad Sajjad, Xi Wei, Zhengying Lu, Ye Wang

**Affiliations:** ^1^State Key Laboratory of Cotton Biology, Institute of Cotton Research of Chinese Academy of Agricultural Sciences, Anyang, China; ^2^Handan Academy of Agricultural Sciences, Handan, China

**Keywords:** cotton, salinity, miRNA, UMI, stress responses

## Abstract

To identify the regulatory network of known and novel microRNAs (miRNAs) and their targets responding to salt stress, a combined analysis of mRNA libraries, small RNA libraries, and degradome libraries were performed. In this study, we used unique molecular identifiers (UMIs), which are more sensitive, accurate, and reproducible than traditional methods of sequencing, to quantify the number of molecules and correct for amplification bias. We identified a total of 312 cotton miRNAs using seedlings at 0, 1, 3, and 6 h after NaCl treatment, including 80 known ghr-miRNAs and 232 novel miRNAs and found 155 miRNAs that displayed significant differential expression under salt stress. Among them, fifty-nine differentially expressed miRNAs were simultaneously induced in two or three tissues, while 66, 11, and 19 were specifically expressed in the roots, leaves, and stems, respectively. It is indicated there were different populations of miRNAs against salt stress in roots, leaves and stems. 399 candidate targets of salt-induced miRNAs showed significant differential expression before and after salt treatment, and 72 targets of 25 miRNAs were verified by degradome sequencing data. Furthermore, the regulatory relationship of miRNA-target gene was validated experimentally via 5′RLM-RACE, proving our data reliability. Gene ontology and KEGG pathway analysis found that salt-responsive miRNA targets among the differentially expressed genes were significantly enriched, and mainly involved in response to the stimulus process and the plant hormone signal transduction pathway. Furthermore, the expression levels of newly identified miRNA mir1 and known miRNAs miR390 and miR393 gradually decreased when subjected to continuous salt stress, while overexpression of these miRNAs both increased sensitivity to salt stress. Those newly identified miRNAs and mRNA pairs were conducive to genetic engineering and better understanding the mechanisms responding to salt stress in cotton.

## Introduction

Cotton (*Gossypium hirsutum* L.) is one of the primary agricultural and cash crops in the world and a major source of fiber and oil. The productivity and quality of cotton are adversely affected by abiotic factors such as soil salinization and land desertification. Salt stress is the most severe and wide-ranging factor that limits cotton growth and influences its biological and metabolic pathways, causing huge losses in cotton fiber and seed output worldwide (Ashraf, 2002; Munns and Tester, 2008). Many studies have already attempted to test the differential expression of genes and miRNAs as they react to salt stress via high-throughput sequencing of transcriptome in cotton and other plant species (Lelandais-Briere et al., 2010; Yang et al., 2020). miRNAs and genes responding to salt stress have the potential for improving environmental adaptation to salinity in plants.

Plants have evolved a range of regulatory mechanisms to avoid or resist salt stress at various phases (Xiong et al., 2002; Zhu Y. et al., 2020). Previous studies proved that a large number of genes play key functions in response to salt stress, leading to a better understanding of their related regulatory mechanisms. For instance, the ABP9 gene encodes a bZIP transcription factor, which overexpression in transgenic *Arabidopsis* plants improved tolerance to drought, salt, and other abiotic stresses (Zhang et al., 2011). Cotton plants overexpressing ABP9 have enhanced tolerance to salt and osmotic stress (Wang et al., 2017). GhMYB73 increases salt tolerance by interacting with PYL8 in cotton and *Arabidopsis* (Zhao et al., 2019). B3 DNA-binding domain proteins and auxin response factor (ARF) are involved in the response to environmental changes (Fedoroff, 2002; Himmelbach et al., 2003). Except for those protein-coding genes, miRNAs, endogenous non-coding RNAs (18–25 nt in length), also play an important role in regulating target genes at the post-transcriptional or translation level (Rae Eden et al., 2013; Dong et al., 2015). miRNAs bind to reverse complementary sequences to form RNA-induced silencing complexes (RISC), which lead to cleavage or translational inhibition of the target RNAs (Axtell, 2013). miRNAs are involved in different biological, physiological, and molecular processes and elucidate the links with plant growth, development, signal transmittal, and tolerance to many biotic and abiotic stresses (Babar et al., 2008; Lu and Huang, 2008; Ma et al., 2021). miR156 increased stress tolerance and maintained the balance of salt stress and development in rice (Long-Gang et al., 2015). miR394 negatively responded to salt stress via modulating LCR expression in *Arabidopsis* (Song et al., 2013). Upregulation expression of miRNA396c increased the sensitivity to salt and alkali stress tolerance in rice (Gao et al., 2010). miRNVL5, a new microRNA, modulated the salt response via regulating GhCHR expression in cotton (Gao et al., 2016). Other miRNAs, including miR159, miR167, miR168, miR171, and miR319, all displayed altered expression levels when exposed to salt stress in plant (Cui et al., 2018; He et al., 2018).

RNA-seq and PCR are important tools for biological research and detecting the expression level of miRNAs. These techniques have two challenges: one is the small copy numbers that limit detection, and the other is the amplification bias that reduces quantitative accuracy, which both reduce quantitative accuracy (Hashimshony et al., 2012). Unique molecular identifiers (UMIs), typically random oligonucleotides ligated to each molecule, are highly sensitive, accurate, and reproducible, and can directly count molecules to correct amplification bias (Baek et al., 2016). The UMI method begins with a reverse-transcription reaction using a primer designed with an anchored polyT and a unique barcode, which enable sequencing reads to be assigned to individual transcript molecules and thus the removal of amplification noise and biases. The quantitative precision of this method is thus improved, especially at low molecule counts (Islam et al., 2011), and UMI method was widely used for high-accuracy (Zhu T. et al., 2020; Karst et al., 2021).

In the study, to get a better comprehension of molecular basis of the salinity stress reaction in cotton, we combined small RNA (sRNA) sequencing with transcriptome and degradome sequencing to identify conserved and novel miRNAs. Three hundred and twelve salt-responsive miRNAs were identified. Some miRNAs, including novel-mir83, ghr-miR167a, novel-mir164, ghr-miR7504a, have high positive correlation with Ghir_D05G03866, Ghir_A08G014350, Ghir_A09G023160, Ghir_D12G015970, and ghr-miR399b/c, ghr-miR2949a-5p, ghr-miR169a, have high negative correlation with Ghir_D10G003030, Ghir_D10G002060, Ghir_A13G020490, respectively. This study uncovering new salt-responsive miRNAs and corresponding targets will enrich the regulatory network of plant resistance to salt stress and facilitates the molecular breeding of cotton.

## Materials and Methods

### Plant Materials and Stress Treatment

The salt-tolerant cotton cultivar “E911” (E911 was provided by the Institute of Cotton Research of Chinese Academy of Agricultural Sciences) was used in this study to test miRNA-target response to salt stress. Seeds were sterilized with 70% (v/v) ethanol for 2 h, and then were washed with sterilized water at least three times. The sterilized seeds were germinated on half-strength Murashige and Skoog (MS) medium (pH 5.8) containing 0.8% agar under a 16 h light/8 h dark cycle. After 3–5 days, better-germinated seedlings were transferred to pots containing an aerated Hoagland nutrient solution (Ahmad et al., 2013). The growth conditions of the seedlings were: 28/20C day/night temperature, 55–70% relative humidity, and a 14/10 h light/dark cycle. At the three-true leaf stage, seedlings showing normal growth were randomly divided into two groups: one was treated with 300 mM salt stress, and experienced salt treatment for 1, 3, and 6 h, and one without salt treatment served as the control. After treating the seedlings with salt, we measured relative electric conductivity and plant water content according to the previously published methods (Yao et al., 2011). For material harvest, the roots, stems, and leaves from the same position on five different plants in each treatment were mixed separately, replicated three times and immediately plunging them in liquid nitrogen and storing them at –80°C for lateral RNA isolation.

### Construction of Small RNA Libraries and RNA Libraries

Before small RNA and mRNA library construction, the total RNA was isolated and purified using TRIzol reagent (Invitrogen, CA, United States) according to the manufacturer’s instructions (Carra et al., 2007). It was then tested using the Agilent 2100 bioanalyzer system to ensure RNA quality. We treated the samples of 1, 3, and 6 h with salt, performing three biological replicates each. In total, 36 small-RNA and RNA-seq libraries were sequenced.

RNAseq is a powerful tool for gene expression pattern analysis in tissues. However, losses in cDNA synthesis and bias in cDNA amplification in RNAseq lead to quantitative errors. miRNA is more likely to produce reading biases than a longer gene with the same transcription level because many miRNAs with the same sequence can be produced from multiple genomic loci. To correct for amplification bias, we used UMI for direct molecular counting. The general process used for constructing the small RNA library is as follows: small RNA 18–30 nt in length was isolated on a 15% polyacrylamide gel and the 5-adenylated, and 3-blocked adaptor were ligated to the 3′ end of the small RNA fragment. UMI (Dai et al., 2014) labeled adaptor (8–10 nt sequences) was ligated to 5′ end, and the unligated adaptor was digested. Purified RNAs were reverse-transcribed to cDNA with UMI labeled primer and the library was validated (Supplementary Figure 1). The miRNA-seq library was sequenced using the BGISEQ-500 platform (Wang et al., 2009).

mRNA-seq libraries were as follows: 5 μg of mRNA was isolated from the total RNA using oligo (dT) magnetic beads (Invitrogen, Carlsbadcity, CA, United States), fragmented and reverse transcribed into cDNA. Adapters with a hairpin loop structure were ligated to cDNA molecules and amplified by PCR. The RNA-seq library was sequenced using the DNBSEQ-T7 platform. All prepared RNA samples were sequenced at the Beijing Genomics Institute (BGI) in Wuhan. Three biological replicates from each sample were used for all experiments.

### Analysis of RNA-Seq and miRNA-Seq Data

To purge the miRNA-seq raw data of low-quality reads, we screened the raw reads from small-RNA libraries (removing of 5′ primer contaminants, no-insert tags, oversized insertion tags, low-quality tags, poly A tags and small tags, and/or tags without 3′ primer) to obtain clean reads. We used the clean sequences to search GenBank^1^ and the Rfam database^2^ to annotate rRNA, tRNA, snRNA, and snoRNA (Gardner et al., 2009; Saiful et al., 2014). After removing sequences of rRNAs, tRNAs, snRNAs, and snoRNAs, the remaining sequences were used in a blast search against miRBase22^3^ to identify conserved, and miRA program to predict novel miRNA by exploring the characteristic hairpin structure of miRNA precursor. Key parameters of miRA were chosen based on an analysis of miRBase annotated miRNAs. Details involving the identification method can be found in Evers et al. (2015).

For miRNA expression, Unique molecular identifier (UMI), 8–10 nt sequences. It was connected to cDNA molecules to marking each molecule in the original sample at the early stage of library construction. It was used to reduce the quantitative bias introduced by PCR amplification, and is conducive to obtaining enough readings for testing. We calculate the species numbers to accurate quantitative of miRNAs. Differentially expressed miRNA proposes a novel method based on the MA-plot (Yang et al., 2002). we defined a gene as a differentially expressed miRNA when reads number fold change ≥ 2 and *Q*-value ≤ 0.001. Plant miRNAs typically have perfect or near-perfect complementarity with their targets, which allows for the identification of targets using TargetFinder and psRobot14 (Fahlgren and Carrington, 2010; Wu et al., 2012), using position-dependent scoring systems to predict miRNA targets.

To obtain RNA-seq clean reads, adapters and low-quality sequence reads were removed from the raw data using SOAPnuke (version v1.4.0) (Chen et al., 2018). These clean reads were mapped to the *Gossypium hirsutum* (AD1, upland cotton), HAU genome^3^ using HISAT2 (version 2.1.0) (Kim et al., 2015; Wang et al., 2017). The gene expression levels in all libraries were presented as fragments per kilobase of transcript per million (FPKM). Differentially expressed genes (DEGs) were defined as those having at least twofold change in expression (false discovery rate, FDR < 0.05).

All data are available in the NCBI SRA database.

### Degradome Sequencing of 5′ RACE Libraries and Data Analysis

Degradome sequencing provides a method for predicting miRNA-target interactions. The libraries were constructed according to the previous description (Romanel et al., 2012). Twenty micrograms of control and salt-treated RNA mixed samples were used for degradome sequencing. Briefly, a 5′RNA adapter was ligated to the cleavage products, which possessed a free phosphate at the 5′end. The purified ligated products were reverse-transcribed to cDNA. After amplification, The *Mme*I enzyme was used to digest the PCR products and ligated to the 3′adapter. The Illumina Genome Analyzer was used to amplify and sequence the final cDNA library, resulting in 49 nt raw reads. After filtering, the clean data were classified by alignment to the database, and non-coding RNAs were removed. Finally, the miRNA–mRNA pairs were identified and the true miRNA cleavage site was mapped to the genes.

### 5′-RLM-RACE

To determine the reliability of degradome sequencing data, a RLM-RACE assay was performed with the RLM-RACE kit (Takara) according to the manufacturer’s instruction. Briefly, total RNA was extracted from cotton leaves. Approximately 2 μg RNA was ligated to the RNA Oligo adaptor (CGACUGGAGCACGAGGACACUGACAUGGACUGAAGG AGUAGAAA), and the ligated RNA was used to synthesize cDNA using M-MLV reverse transcriptase according to the manufacturer’s instructions. Perform RLM-RACE reactions with the adaptor sequence primers and gene-specific primer (GSP1), and gene-specific primer (GSP2). Two rounds of nested PCR were performed for sequencing. The primers used in this assay are listed in the Supplementary Table 6.

### Gene Ontology and Kyoto Encyclopedia of Genes and Genomes Analysis

Bioinformatics analysis uncovered the miRNA-gene regulatory network of biological processes and molecular functions. The functional enrichment analysis was applied to assign gene ontology (GO) annotations, exploring the candidate genes associated with miRNAs, which may play important biological functions in cotton. To perform this analysis, we first maps all genes to GO-terms in the database,^4^ which calculates the gene numbers for every term. The hypergeometric test is then used to find significantly enriched GO-terms in the input gene list. The algorithm is described as follows:


$$P=1-\sum_{i=0}^{m-1}\frac{\left(\begin{array}{c} M\\ i \end{array}\right)\left(\begin{array}{c} N-M\\ n-i \end{array}\right)}{\left(\begin{array}{c} N\\ n \end{array}\right)}$$

N is the number of all genes with GO annotation; n is the number of DESs target genes in N; M is the number of all genes that are annotated to a specific GO term; m is the number of DESs target genes in M. The *P*-value is corrected by using the Bonferroni method, a corrected *P*-value ≤ 0.05 is taken as a threshold. GO terms fulfilling this condition are defined as significantly enriched GO terms. Similarly, The Kyoto Encyclopedia of Genes and Genomes (KEGG) database was used to assign the pathway annotations.^5^ Gene numbers for every GO term, pathway, and hyper-geometric distribution were used to detect significantly enriched GO terms and pathways with a *P*-value threshold of ≤ 0.05.

### Differential Expression Analysis

UMI was connected to cDNA molecules by marking each molecule in the original sample at the early stage of library construction. It was used to reduce the quantitative bias introduced by PCR amplification and is conducive to obtaining sufficient readings for testing. We calculated the species numbers for accurate quantitative comparison of sRNAs. The expression levels of known miRNAs in each library were calculated and the miRNA expression levels between different samples were compared.

DEGseq (Hafner et al., 2008) is a method based on the MA-plot, which is a statistical analysis tool widely used to detect and visualize intensity-dependent ratio of microarray data (Li et al., 2010). To improve the accuracy of the DEG results, we defined a gene as a DEG (differentially expressed gene) when the number fold change was ≥ 2 and the *Q*-value was ≤ 0.001.

### Association Analysis of miRNAs and mRNAs

Pearson correlation coefficients (r values) between expression rates of small RNA and mRNA were constructed and calculated by Graph Pad Prism version 6, with two-tailed *t*-tests. *P*-values < 0.05 were considered statistically significant. We screened the differentially expressed microRNAs and their predicted target genes. Based on the difference between processed and control samples, the interaction between the miRNA and the targeting relationship was shown with VisNetwork.

### Plasmid Construction and Transformation of Cotton

For cotton transformation, the pCLCrV:VIGS construct was constructed using a method modified from Ye et al. (2014). The fragment of pre-miRNA was synthesized (Sangon) and inserted into the plant expression vector pCLCrVB after the CaMV35S promoter using *Bam*HI and *Sac*I. The vector was introduced into the Agrobacterium tumefaciens strain GV3101. The Agrobacterium cultures were pelleted and resuspended. After 3 h incubation at room temperature, Agrobacterium harboring pCLCrVA was mixed with an equal volume of Agrobacterium harboring pCLCrVB. The mixed Agrobacterium solutions were infiltrated into the abaxial side of cotyledons of 2-week-old cotton seedlings using needleless syringes through small wounds which were made on the surface of cotyledons or true leaves using small syringe needles. Keep the plants at 24°C and shield them from light for 48 h, then were grown in a controlled environment at 28°C under long-day conditions (16 h light/8 h dark) with white light illumination for about half a month, finally treated with 300 mM NaCl solution.

### Measurement of Relative Electric Conductivity and Malondialdehyde

The gene silenced plants and control plants CLCrV:00 were used for salt stress treatment. The functional leaves (3 plants each treatment, three repeats) were collected in each treatment for the measurements of relative electric conductivity (REC), malondialdehyde (MDA). The MDA content were measured by using the maleic dialdehyde assay kit (the Nanjing Jiancheng Institute in Najing, Jiangsu, China). The leaf REC was determined as described previously (Yu et al., 2006).

## Results

### Phenotypic Analysis of Salt-Tolerant Cotton Cultivar E911 to Salt Treatment

**FIGURE 1:**
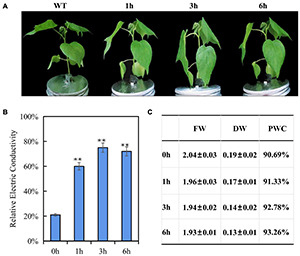
Phenotypic and physiological changes of three-leaf-stage cotton under salt stress. **(A)** Performance of cotton seedlings under salt stresses for 0, 1, 3, 6 h. **(B)** Relative Electric Conductivity (REC), each bar value represents the mean ± SD of three independent experiments; ^∗∗^ indicates *P*-value < 0.01. **(C)** Effects of salt stress on plant water content of E911. FD, Fresh weight; DW, Dry weight; PWC, Plant water content. Three plants with one replicate are used to evaluate the FD, DW, and PWC. Three replicates were performed.

Cotton seedlings of three-true leaf were treated with 200 mM NaCl, no visible phenotype change was observed within 6 h (Supplementary Figure 2). After a 300 mM NaCl solution treatment, the leaves showed mild dehydration phenotype (leaf and petiole were slightly bent downward) at 1 h, and severe dehydration phenotype (leaf and petiole were strongly bent downward) at 3 h, but less dehydration phenotype (leaf and petiole were moderately bent downward) at 6 h than 3 h (Figure 1A), a hypothesis is that 300 mM NaCl treatment may contribute to induce a salt-tolerant phenotype at later development stage due to early triggering an inherent immune response to salt stress. For testing this hypothesis, 400 mM NaCl treatment was performed, the seedling presented dehydration phenotype (leaves droop down) after 1 h treatment, and became more and more severe dehydration (Leaf drooping is more serious, and the bottom leaves are curled) with the increase of treatment time, suggesting that 400 mM NaCl produced the irreversible damage and the seedlings will die finally (Supplementary Figure 2). According to phenotypic changes, 300 mM NaCl was chosen for the further experiments. As expected, the electric conductivity in leaves was significantly increased at three time points under the 300 mM NaCl treatment as compared to the control and reached the peak after 3 h, but with decreased electric conductivity at 6 than 3 h (Figure 1B); fresh weight and dry weight of seedling leaves decreased gradually and finally stabilized along with the increased salt treatment time, while plant water content increased gradually (Figure 1C), the changed trend of electric conductivity, fresh weight and dry weight both consisted with the wilting phenotypes of rising first and then falling after 300 mM NaCl treatment. These results indicated that fast response in E911 was occurred after salt treatment. Therefore, these samples (0, 1, 3, and 6 h after salt treatment) were used to perform miRNA-seq and RNA-seq analysis to understand the salt stress response mechanism in cotton. For miRNA-seq and RNA-seq data, we measured the correlation coefficient of gene expression among all samples, the Pearson correlation of biological repetition is higher. Therefore, the repeatability among replicates and the validity of data were confirmed (Supplementary Figures 2, 3).

### Dynamic Patterns of miRNAs Composition in Root, Stem, and Leaf

**FIGURE 2:**
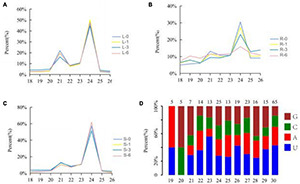
Percentages of the small RNAs with 18–26 nt lengths to the total miRNAs. Y-axis represents percentages of miRNAs identified in this study; X-axis represents the length of miRNAs. **(A)** Leaf; **(B)** root; **(C)** stem. Blue, yellow, red, and green lines represent 0, 1, 3, and 6 h, respectively. **(D)** The first base distribution of predicted miRNAs.19–30 represents the length of miRNA; 5, 5, 7, 14, etc. represents the number of miRNA.

A total of 1,107,343,118 reads were obtained from the twelve cotton small RNA libraries generated from the salinity treatment and the control tissues. Of 80.37% were clean reads, including miRNA, rRNA, snRNA, snoRNA, tRNA, degraded fragments of mRNA introns or exons and several other annotated reads, ranging from 18 to 44 nt in length. The sequences 18–28 nt in length, which accounted for 70.31% of all the clean reads, were extracted to analyze the distribution of small RNA length and the first base distribution, found that the first base at the 5′ end of microRNA of 21–23 nt has a strong preference for U, which coincided with previous study about the miRNA characterizes, suggesting the data is available (Figure 2). The length of the small RNAs was not evenly distributed in each library, while a high incidence of 21–24 nt sequences was observed. This accounted for 53.97% of the total clean reads, and represents the typical length of mature miRNAs in plants. The number of 24 nt sequences significantly exceeded that of other sequences, followed by 21 nt (Figures 2A–C). Compared with leaves and stems, the percentage of 18–26 nt miRNAs, especially 21 and 24 nt miRNAs, fluctuated between the control (0 h sample) and other time points in the root, where the number of 21 and 24 nt miRNAs decreased, the others increased when the root was subjected to salt stress (Figures 2A–C). We believed that the root responds most directly to salt stress since more miRNAs with different lengths were involved in the response to salt stress in the root than the stem and leaves.

### Identification of Conserved and Novel miRNAs

In order to avoid the deviation of sequence composition caused by species differences, we collected only known cotton miRNAs in the central miRNA Registry Database miRBase for miRNA-seq analysis. Statistical results of every library showed that compositions of siRNA families is similar in the CK, 1, 3, and 6 h salt-treatment samples, including rRNA (∼1.83, 2.52, 3.33, and 3.10% for the unique reads), snRNA (∼0.03, 0.07, 0.07, and 0.04% for the unique reads), snoRNA (∼0.03, 0.05, 0.06, and 0.05% for the unique reads), and tRNA (∼0.31, 0.40, 0.52, and 0.42% for the unique reads) (Table 1). miRNAs that differed by only two mismatches from known miRNAs were characterized as conserved miRNAs within miRBase. The miRNA genes primarily originate from independent transcriptional units, so RNAseq was performed to identify primary transcripts of novel miRNAs. Total reads were derived from 12 sRNA-seq libraries from CK, 1, 3, and 6 h salt-treatment tissues (three biological replicates per treatment). After cleaning the low-quality reads, 2090.3 M of 2490.2M clean reads were mapped to genome and a total of 422.2M unigenes were identified. The data of RNAseq was used for mapping reads and predicting the hairpin structure of precursors of miRNAs. Each miRNA-precursor had high minimal folding free energy index (MFEI) values. As a result, a total of 80 known ghr-miRNAs and 232 novel miRNAs in salt treated root, stem and leaf were identified in all libraries (Supplementary Table 1).

**TABLE 1:** Dataset summary of small RNAs from different salt-treatment tissues.

**Category**	**WT**	**1 h**	**3 h**	**6 h**
Total reads	258,594,758	273,103,499	297,933,959	277,710,902
High quality	255,979,918	270,838,290	295,260,105	275,601,191
PloyA	7,024	8,461	6,277	8,227
Invalid	6,820,607	7,961,725	10,141,136	6,377,366
Clean reads	222,965,736	224,541,353	219,152,920	223,053,752
Unique sRNAs	213,062,991	211,618,778	209,845,637	212,424,881
Mapped to genome	179,572,503 (84.28%)	192,924,234 (90.55%)	183,927,175 (86.33%)	196,629,412 (92.29%)
Exon	3,981,290 (1.87%)	4,539,444 (2.13%)	5,370,304 (2.52%)	5,422,106 (2.54%)
miRNA	1,341,383 (0.63%)	1,140,948 (0.54%)	997,800 (0.47%)	1,067,406 (0.50%)
snoRNA	66,721 (0.03%)	101,831 (0.05%)	131,388 (0.06%)	105,628 (0.05%)
tRNA	655,924 (0.31%)	844,016 (0.40%)	1,099,397 (0.52%)	887,019 (0.42%)
snRNA	74,014 (0.03%)	140,876 (0.07%)	155,570 (0.07%)	86,163 (0.04%)
rRNA	3,896,863 (1.83%)	5,376,499 (2.52%)	7,091,959 (3.33%)	6,605,936 (3.10%)

### Identification of Regulatory Networks Consist of miRNAs and Targets Responding to Salt Stress in Cotton

**FIGURE 3:**
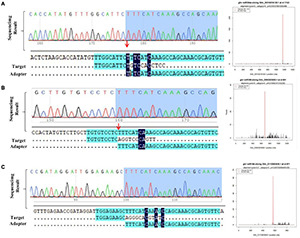
Detection of target gene cleaved site of miRNA. **(A–C)** T-plot validation of predicted target mRNAs of conserved salt stress-responsive miRNAs by degradome sequencing and 5′RLM-RACE experiment.

A total of 1,662 putative targets of 312 miRNAs were identified through above methods. To further verify the predicted miRNA-target interactions, a degradome library was constructed, which generated 28.31M sequence reads. After consecutive steps of filtering, 27.55M reads were obtained, and processed for identification of cleavage sites. Through degradome data analysis, a total of 72 genes were identified as the potential targets of 25 miRNAs (Supplementary Table 2). As mentioned in previous studies, we identified some known salt responsive miRNA and targets including Ghr-miR164-Ghir_A01G003240 (NAC domain-containing protein 21/22, NAC transcription factor) (Ganesan et al., 2008), Ghr-miR166b-Ghir_D13G025080 (Homeobox-leucine zipper protein REVOLUTA, HD-ZIP transcription factor) (Shin et al., 2004), Ghr-miR393-Ghir_D11G006890 (Protein AUXIN SIGNALING F-BOX 2) (Chen et al., 2015), Ghr-miR172-Ghir_A10G021190 (Ethylene-responsive transcription factor RAP2-7, ERF transcription factor) (Shi et al., 2015). To further detect our data reliability, randomly selected miRNA-targets pairs including Ghr-miR394-Ghir_ D01G016120, Ghr-miR398-Ghir_D05G023600, Ghr-miR164-Ghir_D11G003630 and Ghr-miR482a-Ghir_A11G011080 were experimentally detected via 5′RLM-RACE assay. The sequenced RACE products verified the cleavage relationship of 3 pairs of miRNA-targets, proving the availability of degardome data (Figures 3A–C). Salt stress-responsive miRNAs (from small RNA-seq) and their target genes (from RNA-seq) were integrated to better understand the role of miRNAs. Correlation analysis of miRNA and their target mRNA expression profiles using Pearson’s correlation coefficient (*r* > 0.6) identified a total of 147 miRNA-mRNA interaction pairs under salt stress conditions. These correlation pairs were comprised of 126 genes and 38 miRNAs (Supplementary Table 3). Fifty-six pairs displayed a positive correlation and 91 displayed a negative correlation. Multiple potential target genes of the conserved miRNA ghr_miR7504a/b and novel miRNAs novel_mir83 were listed (Supplementary Figure 4), which showed significant differential expression under salt stress. These novel regulatory networks consisting of miRNA and the corresponding target will help better understand how salt stress is regulated in cotton.

### Salt-Responsive miRNAs Have Tissue- Conservative or Specific Functions

**FIGURE 4:**
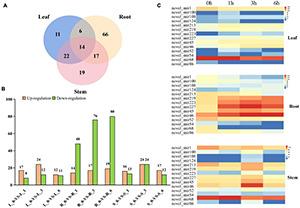
Differentially expressed miRNAs and cluster analysis. **(A)** Number of different DE miRNAs in leaves, roots and stems. **(B)** The number of DE miRNAs in leaves, stems, and roots under control and different time points. The number of up-and down-regulated genes are depicted in the form of bars, respectively. **(C)** The expression patterns of those novel miRNAs in leaf, stem and root.

The abundance of the identified miRNAs in the 12 libraries ranged from 0 to 470,902, indicating that the expression of the miRNAs varied greatly. In order to identify miRNAs that are responsive to salt stress, expression patterns and WGCNA of miRNAs were performed. First, we analyzed the relative expression levels of miRNAs in response to salt stress at different time points among root, stem and leaf. A total of 155 miRNAs showed significant differentially expressed patterns under salt stress (Figure 4A and Supplementary Table 4). Among them, 6 differentially expressed (DE) miRNAs were observed in the root and leaves, 17 DE miRNAs were detected in the roots and stem, 22 DE miRNAs were simultaneously induced in the leaves and stem, and 14 DE miRNAs were existed in all of the three organs. In addition to conserved DE miRNAs in three tissues, 66, 11, and 19 miRNAs were expressed specifically in the root, leaves, and stems, respectively (Figure 4A and Supplementary Figure 5). A conclusion is that miRNAs responding to salt stress have conservation and tissue specificity.

There is significantly more differentially expressed (DE) miRNAs in the roots than in the leaves and stems at 1, 3, and 6 h after salt treatment (Figure 4B). As time passed, the number of DE miRNAs also significantly increased in roots not in stem and leaf (Figure 4B and Supplementary Figure 5), suggesting that the root responds most directly to salt stress. In addition of the number difference of DE miRNAs in root, stem and leaf, the expression pattern also had huge differences. Compared with 0 h, most DE miRNAs in roots were downregulated after salt treatment, while similar number of up-regulated and down-regulated miRNAs were detected in stem and leaf (Figure 4B). Interestingly, miRNAs that are differentially expressed simultaneously in root, stem and leaf had different basal expression level at 0 h, but most of these miRNAs presented similar induction patterns after salt treatment (Figure 4C), supporting that conserved miRNAs in different tissues may have conserved function. The expression level of miR393 and its target gene (Ghir_D11G006890) both has significantly changed in root not stem and leaf, indicated miR393 specifically regulates salt stress through Ghir_D11G006890 in roots (Supplementary Figure 6).

**FIGURE 5:**
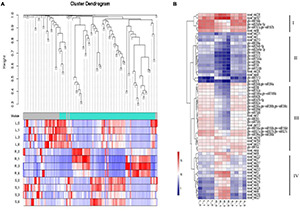
Expression patterns of miRNA targets involved in stress response and hormone pathway. **(A)** Clustering dendrogram of differentially expressed miRNAs. **(B)** Clustering of miRNA expression profiles. The heat map diagram of miRNA expression was prepared with hierarchical clustering of miRNA expression in different tissues at different times. miRNAs are shown in the rows and each column represents a sample. The miRNA clustering tree is shown on the right (clusters I, II, III, and IV). L, Leaf; R, Root; S, Stem.

For miRNA co-expression network, 155 differentially expressed miRNAs and 12 samples were subjected to WGCNA analysis. After filtering out the genes with a low expression (FPKM < 0.05), coexpression networks were constructed on the basis of pairwise correlations of gene expression across all samples. Modules were defined as clusters of highly interconnected genes, and genes within the same cluster have high correlation coefficients. This analysis identified 2 distinct modules (labeled with two different colors), which correlated with distinct samples due to sample-specific expression profiles (Figure 5A). We chose DE miRNAs in different tissues for a cluster analysis. Four clusters were constructed based on the expression patterns of response to different stress conditions in the root, leaf, and stem tissues (Figure 5B). Cluster I contains miRNAs with high expression in roots, stems, and leaves; Cluster II shows low expression in roots, stems, and leaves. Most miRNAs with lower expression levels in the root were categorized into cluster III; most miRNAs with higher expression levels in the root were categorized into cluster IV. Collectively, salt-responsive miRNAs displayed unique regulation mechanisms, where miRNAs have tissue-conservative or tissue-specific expression pattern responding to salt stress.

### Gene Ontology and Kyoto Encyclopedia of Genes and Genomes Pathway Analysis Among Targets of Salt-Induced miRNAs

**FIGURE 6:**
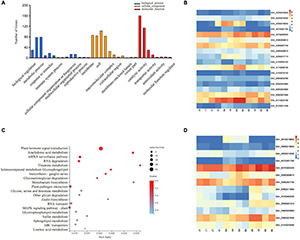
Go and KEGG pathway analysis of differentially expressed miRNAs targets. **(A)** Gene Ontology classification Analysis of differentially expressed miRNA targets. **(B)** The expression pattern of DEGs involved in the stimulus response. **(C)** Scatter plot of most enriched KEGG pathways of DEGs. Gene ratio represents the ratio of the number of DEGs in the pathway and total number of DEGs with pathway annotation. **(D)** The expression patterns of DEG involved in plant hormone signal transduction. DEGs, differentially expressed genes.

GO-based analysis determines which GO terms (biological process, molecular function, and cellular component) a gene belongs to Eilbeck et al. (2005). Therefore, GO-based analysis can provide more insight into understanding the function of the miRNA targets. Of the significantly up- and down-regulated differentially expressed (DE) genes, 399 targets of salt induced miRNAs were selected for Gene Ontology classification analysis. We obtained 33 GO terms including 14 biological process, 11 cellular components, and 8 molecular functions. Further analysis found that biological processes related to biological regulation, cellular process, metabolic process, and response to stimulus were significantly overrepresented. Molecular functions including binding, catalytic activity, transcription regulator activity and transport activity were all activated (Figure 6A). Notably, among the above-mentioned 399 genes, 18 genes were associated with the biological process of stimulus response, and 13 genes were involved in hormone signal transduction (Figures 6B,D). Further analysis supports that these genes were involved in plant responses to abiotic stimuli, external stimuli, chemical stimuli, defense response, and hormone stimuli, all of which are likely associated with resistance. KEGG enrichment analysis suggested that the mRNA surveillance pathway (ko03015), RNA degradation (ko03018), RNA transport (ko03013), and Plant hormone signal transduction (ko04075) were the most enriched pathways for salt stress induced miRNA targets (Figure 6C). Among those, the mRNA surveillance pathway, RNA degradation, and RNA transport are closely related to the degradation process of miRNA. Overall, target genes of salt induced miRNAs played vital role in responding to salt stress.

### Overexpressing Salt-Induced miRNAs Altered the Salt Tolerance in Cotton

**FIGURE 7:**
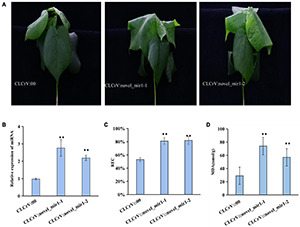
The overexpression of novel_mir1 in cotton increased the sensitivity to salt stress. **(A)** Phenotypes on cotton plant inoculated with CLCrV:00 (empty vector), CLCrV:novel_mir1 after 300 mM NaCl treatment for 6 h. Two independent silencing lines (novel_mir1-1-and novel_mir1-2) are shown. **(B)** Relative expression of miRNA (novel_mir1) in leaves of cotton plants inoculated with CLCrV:00 or CLCrV:miRNA (novel_mir1) were determined through quantitative reverse transcription PCR. **(C)** Relative electric conductivity in leaves of cotton as described in **(A)**. **(D)** Determination of malondialdehyde content in leaves of cotton as described in **(A)**. **p < 0.01.

To explore whether the differentially expressed miRNAs were involved in plant response to salt stress, one novel identified miRNA novel-mir1 and two known miRNAs miR393 and miR390 were selected to overexpress in cotton using the VIGS vectors. We then quantified the expression level of mir1, miR393 and miR390 in CLCrV:novel-mir1, CLCrV:miR393, CLCrV:miR390 plants, respectively, and found a significant increase compared to CLCrV:00 (Figure 7B and Supplementary Figure 7). And compared to the control CLCrV:00, the leaves of the cotton infected with CLCrV:mir1 obviously wilted at 6h after salt treatment under hydroponic condition (Figure 7A). Compared with the control CLCrV:00, the cotton infected with CLCrV:miR393 or CLCrV:miR390 showed yellow or necrotic leaves at 10-day after salt treatment under soil condition (Supplementary Figure 7). To investigate the potential physiological mechanisms by which miR390, miR393, and mir1 regulated plant tolerance, we detected malondialdehyde (MDA) and relative electric conductivity in CLCrV:miR390, CLCrV:miR393, CLCrV:novel-mir1, and CLCrV:00 plants under stress treatment conditions. The results showed that the MDA content, and relative electric conductivity of the CLCrV:miR390, CLCrV:miR393, CLCrV:mir1 was both significantly higher than that of CLCrV:00 (Figures 7C,D and Supplementary Figure 7). These results indicate that known miR390, miR393, and novel identified miRNA novel-mir1 both negatively regulated the response to salt stress in cotton, supporting that the identified salt-induced miRNAs were available, and will enrich the regulatory network of cotton responding to salt stress.

## Discussion

Some miRNAs are activated or inhibited to regulate the downstream targets in plants when exposed to environmental stress (Jones-Rhoades and Bartel, 2004; Khraiwesh et al., 2012). For example, high osmotic stress decreased the expression of miR167a, whereas IAA-Ala Resistant 3 (IAR3), a target of miR167a, showed increased levels of expression when responding to drought tolerance (Natsuko et al., 2012). The expression level of osa-MIR393 is down-regulated under salinity stress, overexpression of osa-MIR393 in transgenic rice and *Arabidopsis* was more susceptible to salt stress (Peng et al., 2011). Over-expressing osaMIR396c showed reduced salt and stress tolerance in plants (Peng et al., 2010). miR399f participates in plant responses to abiotic stresses, especially salt and drought (Baek et al., 2016). miR417 negatively regulates seed germination in *Arabidopsis* under salt stress conditions (Jung and Kang, 2007). Increasing evidence indicates that miRNAs play important roles in plant response to abiotic stress. Target prediction, as well as degradome sequencing data, revealed that a set of cotton miRNAs are allied with these top-poster genes including miR167-IAR3, miR172-AP2, and miR164-NAC (Millar and Frank, 2005; Man et al., 2014). Similarly, our degradome sequencing data and 5′RLM-RACE assay also confirmed those stress-related miRNA targets, including MYB and NAC genes in cotton, supporting our data availability. Moreover, novel identified mir1 negatively regulates response to salt stress when overexpressed in cotton, supporting the idea that the novel miRNAs identified in our research serve key roles in regulating cotton salt resistance.

UMIs are random oligonucleotide barcodes that are increasingly used in high-throughput sequencing experiments. This method corrects for PCR errors and provides an absolute scale of measurement with a defined zero level (Fu et al., 2014). The accuracy of datasets with a low abundance of RNA was significantly improved (Yu F. et al., 2018). UMI-ATAC-seq technique was integrated into standard ATAC-seq procedures, which can rescue about 20% of reads that are mistaken as PCR duplicates in standard ATAC-seq (Zhu T. et al., 2020). In this study, we counted molecules by the total number of distinct UMIs to quantitatively assess miRNA-seq data, which provided accurate expression patterns of miRNA response to salt stress in cotton. Compared with the traditional method, we obtained 14 novel miRNAs via UMIs (Supplementary Table 5). The UMI method allowed us to identify novel miRNAs that enriched the regulatory network of salt response in cotton.

Based on the results of the analysis of the small RNA library, we found that expression patterns of some salt-responsive miRNAs depended on the tissue specialty, where 66, 11, and 19 miRNAs were found to be specifically expressed under salt stress in the roots, leaves, and stems, respectively. Similar studies demonstrated tissue-specific expression patterns in small RNAs in maize and human tissues (Sakaue et al., 2018; Yu X. et al., 2018). Compared with previously reported salt-responsive miRNAs in cotton, our research not only identified conserved miRNAs^6^ (miR164, miR167, miR399, miR7052, etc.), but also novel miRNAs (mir45, mir46, mir223, mir227, mir1, mir68, mir86, etc.), both of which exhibited significant differential expression under salt stress. Compared with the 0 h samples, mir1, mir68, and mir86 showed significant down-regulation at 1, 3, and 6 h; mir45, mir46, mir223 and mir227 showed up-regulation at 1, 3, and 6 h, indicating that different miRNAs functions differently when responding to salt stress. A total of 399 target genes of salt-induced miRNAs showed differential expression patterns in different tissues at different time points, and 147 miRNA-mRNA interaction pairs (comprised of 126 genes and 38 miRNAs) were identified. These novel regulatory networks consist of miRNAs and the corresponding targets will help understand the regulatory mechanisms of cotton response to salt stress.

**FIGURE 8:**
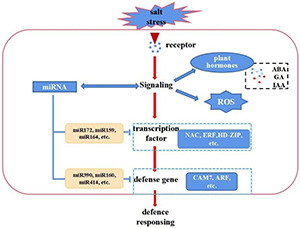
A hypothetical scheme showing a summary of the cascades of various physiological and biochemical events incurred with cotton in resistant genotypes. Upon salt stresses, cellular homeostasis is sensed by a sensory mechanism. The ROS, and hormone signaling cascades are activated. This leads to changes in gene expression which involves transcriptional factors and miRNAs, resulting in changes the expression of regulators (an miR172, miR164, miR390, miR414, etc. that regulate NAC, ERF, HD-ZIP, and CAM7, etc. gene expression are shown as an example).

Cotton firstly senses salt stress signals and subsequently activates or inhibits stress-responsive genes and miRNAs. It also undergoes distinctive biochemical changes, such as ion flux through plasma membranes and the formation of reactive oxygen species (ROS), these results revealed a complex network of miRNAs, and mRNAs under salt stress. On the other hand, increasing evidence suggests that miRNAs play important roles in the regulation of gene expressions. A schematic model was proposed based on this study. However, the functions of some miRNAs were not fully demonstrated and need to be further studied (Figure 8). The salt defense mechanism involves several transcription factors (TFs) like MYB, WRKY, NAC and various hormones including ABA, SA and IAA (Kim et al., 2013; Liu et al., 2014; Zhao et al., 2019). In this study, we performed a comprehensive analysis via integrating mRNA-seq data, miRNA-seq data and degradome data. First, we analyzed the number of DE genes among different tissues and found they gradually increase with the processing time in the leaves, stems, and roots (Supplementary Figure 8A), which coincides with the increase of DE miRNAs. Among these, bHLH (247), AP2 (392), MYB (419), NAC (426), and WRKY (153) were the most overrepresented TF families. Compared with other TFs, the number of NAC families displayed large differences among the roots, stems, and leaves, and was enriched in the roots and stems. This indicates that NAC primarily responded to salt stress in roots and stems (Supplementary Figure 8B). Furthermore, we identified several TFs including NAC, ERF, HD-ZIP, plant hormones related genes, and ROS scavenging-related genes, as targets for salt-responsive miRNAs responding to salt stress (Supplementary Figure 8 and Figure 6). The corresponding target genes CIPK21, CAM7, and CDPK9 of miR414, rsa-mir3, and rsa-mir5 were reported to cooperatively perform their functions in plant responses to abiotic stresses (Ahmad et al., 2013). These identified miRNAs and corresponding targets may contribute to the salinity tolerance of E911 (salt-tolerant variety).

In conclusion, we integrated the miRNA and mRNA data to identify the regulatory network responding to salt stress in cotton. The candidate salt-responsive miRNA and corresponding target genes identified by the UMI method contain both previously reported salt-responsive miRNAs and novel miRNAs that can be used for genetic engineering in cotton. The identified miRNA-mRNA pairs could enrich regulatory mechanisms of cotton salt tolerance.

## Conclusion

In this study, mRNA libraries, small RNA libraries, and degradome libraries were integrated to systematically investigate the response of miRNAs of a salt-tolerant cotton variety to salt stress by UMIs. 59 differentially expressed (DE) miRNAs were simultaneously induced in above two or three tissues, while 66, 11, 19 miRNAs were specially expressed in root, leaf, and stem, respectively, supporting that these miRNAs have conservative and tissue specific function. Furthermore, overexpression of mir1 increased the sensitivity to salt stress, supporting our data is reliable. The identification of these small RNAs as well as elucidating their functional significance broadens our understanding of post-transcriptional gene regulation in response to salt stress.

## Data Availability Statement

The data presented in the study are deposited in the NCBI repository, accession number PRJNA766312.

## Author Contributions

JZ, YD, and YW designed the experiments and drafted the manuscript. XW prepared samples for small RNA sequencing. JZ, GY, and MS performed the high-throughput sequencing data analysis. JZ, GY, and YW contributed to the design and discussion of the work, and assisted in drafting the manuscript. All authors have read and agreed to the published version of the manuscript.

## Conflict of Interest

The authors declare that the research was conducted in the absence of any commercial or financial relationships that could be construed as a potential conflict of interest.

## Publisher’s Note

All claims expressed in this article are solely those of the authors and do not necessarily represent those of their affiliated organizations, or those of the publisher, the editors and the reviewers. Any product that may be evaluated in this article, or claim that may be made by its manufacturer, is not guaranteed or endorsed by the publisher.

## Footnotes

1
https://www.ncbi.nlm.nih.gov/genbank/

2
http://rfam.sanger.ac.uk/

3
https://cottonfgd.org/about/download.html

4
http://www.geneontology.org/

5
http://www.genome.jp/kegg/

6
http://www.mirbase.org

## Supplementary Material

Supplementary Figure 1 Experimental Pipeline Steps for Small RNA Sequencing. (1) Small RNA enrichment and purification. (2) 3′ end adaptor ligation: Ligate the 5-adenylated and 3-blocked adaptor to the 3′ end of the small RNA fragment. (3) Add unique molecular identifiers (UMI) labeled Primer. (4) Unligated adaptors digestion. (5) 5′ end adaptor ligation. (6) First strand synthesis with unique molecular identifiers (UMI) labeled Primer. (7) Second strand synthesis. (8) Fragment selection. (9) Library quantitative and pooling cyclization. (10) Library QC. (11) Sequencing on BGISEQ0000.

Supplementary Figure 2 Correlation analysis of all miRNA-seq samples. Both X and Y axis represent each sample. Coloring indicate Pearson correlation (high: blue; low: white).

Supplementary Figure 3 Correlation analysis of all RNA-seq samples. Both X and Y axis represent each sample. Coloring indicate Pearson correlation (high: blue; low: white).

Supplementary Figure 4 Vis Network of the interaction. Positive and negative regulation relationships are expressed in red and blue, respectively.

Supplementary Figure 5 Heat map of the expression patterns of specific differential expressed miRNAs in different tissues. **(A)** miRNA differentially expressed only in leaves. **(B)** miRNA differentially expressed only in stems. **(C)** miRNA differentially expressed only in roots.

Supplementary Figure 6 The expression patterns of miR393 and its target.

Supplementary Figure 7 **(A,B)** Phenotypes on cotton plant CLCrV:miR390, CLCrV:miR393 after NaCl treatment. **(C)** Relative expression of miRNA (miR390, miR393) in leaves of cotton plants. **(D)** Determination of malondialdehyde content in leaves of cotton. **(E)** Relative electric conductivity in leaves of cotton.

Supplementary Figure 8 **(A)** Number of differential expression genes in different organs. **(B)** Number of differentially expressed TF families in leaves, roots, stems.
