# Supplementary figures and images for: Integration of mRNA and miRNA Analysis Reveals the Molecular Mechanism of Cotton Response to Salt Stress
Source: Front Plant Sci. 2021 Dec 9;12:767984. doi: 10.3389/fpls.2021.767984 (PMC8695560; doi:10.3389/fpls.2021.767984)

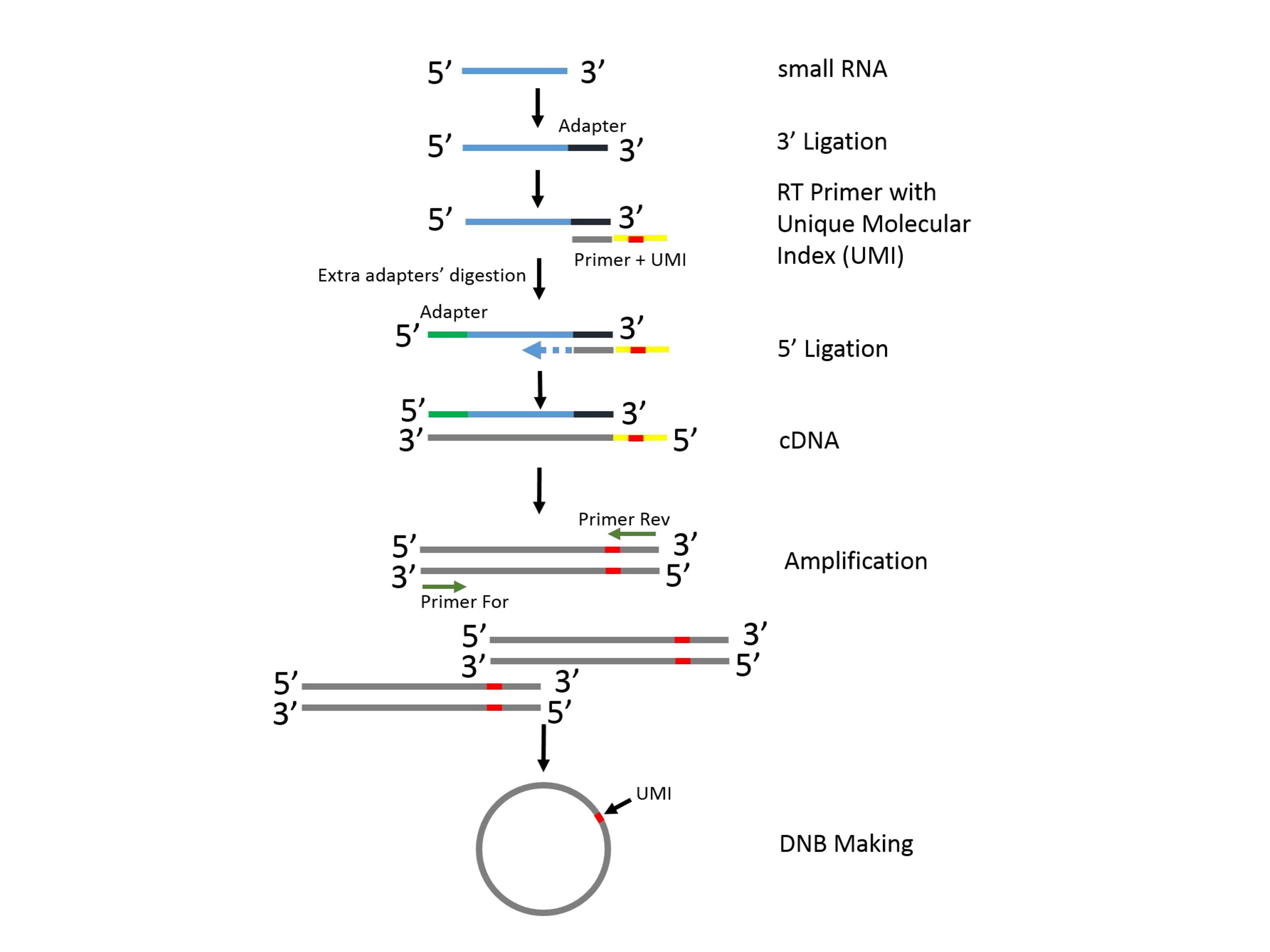

Supplement: Supplementary Figure 1 — Experimental Pipeline Steps for Small RNA Sequencing. (1) Small RNA enrichment and purification. (2) 3′ end adaptor ligation: Ligate the 5-adenylated and 3-blocked adaptor to the 3′ end of the small RNA fragment. (3) Add unique molecular identifiers (UMI) labeled Primer. (4) Unligated adaptors digestion. (5) 5′ end adaptor ligation. (6) First strand synthesis with unique molecular identifiers (UMI) labeled Primer. (7) Second strand synthesis. (8) Fragment selection. (9) Library quantitative and pooling cyclization. (10) Library QC. (11) Sequencing on BGISEQ0000. [file Image_1.JPEG]

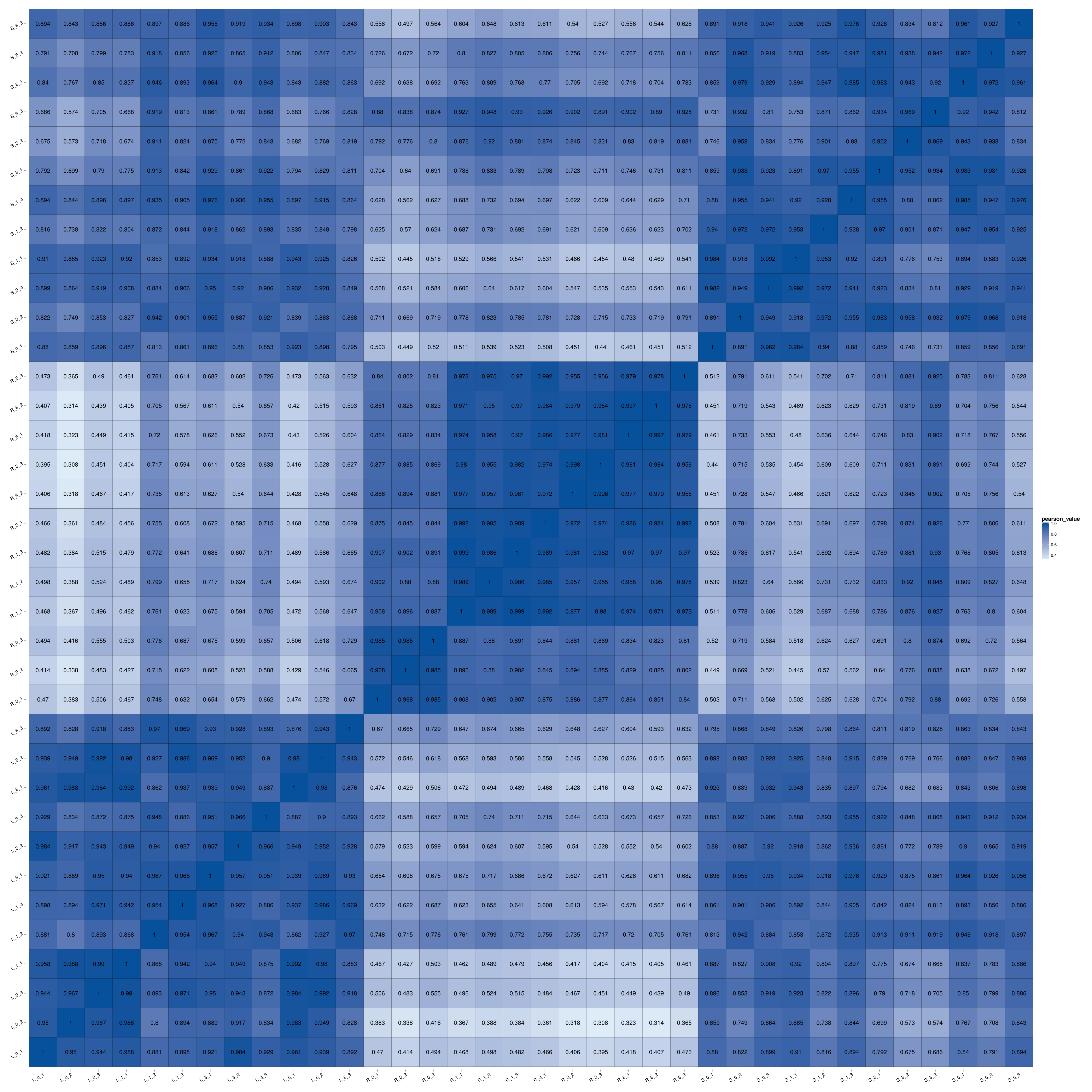

Supplement: Supplementary Figure 2 — Correlation analysis of all miRNA-seq samples. Both X and Y axis represent each sample. Coloring indicate Pearson correlation (high: blue; low: white). [file Image_2.PNG]

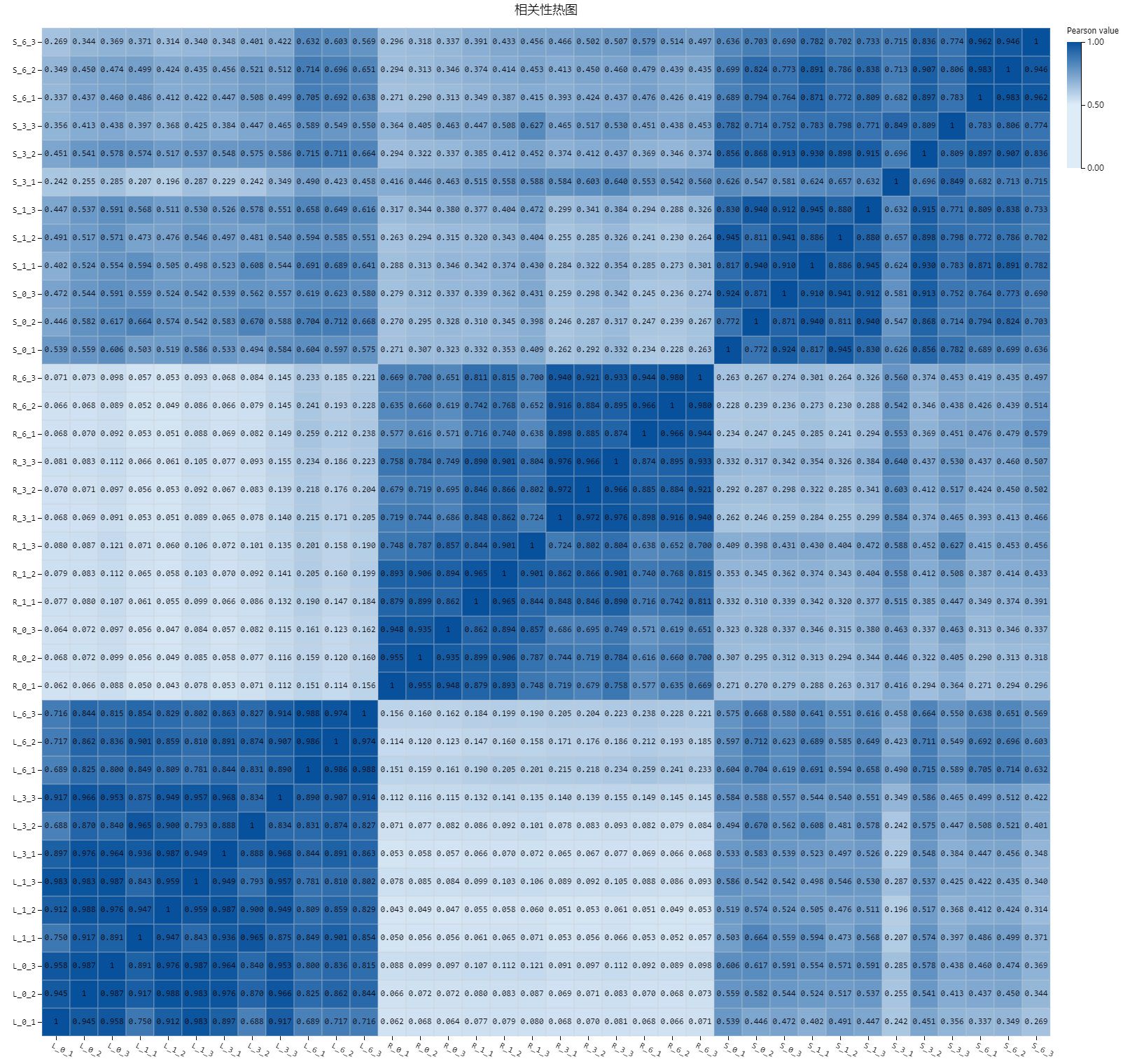

Supplement: Supplementary Figure 3 — Correlation analysis of all RNA-seq samples. Both X and Y axis represent each sample. Coloring indicate Pearson correlation (high: blue; low: white). [file Image_3.JPEG]

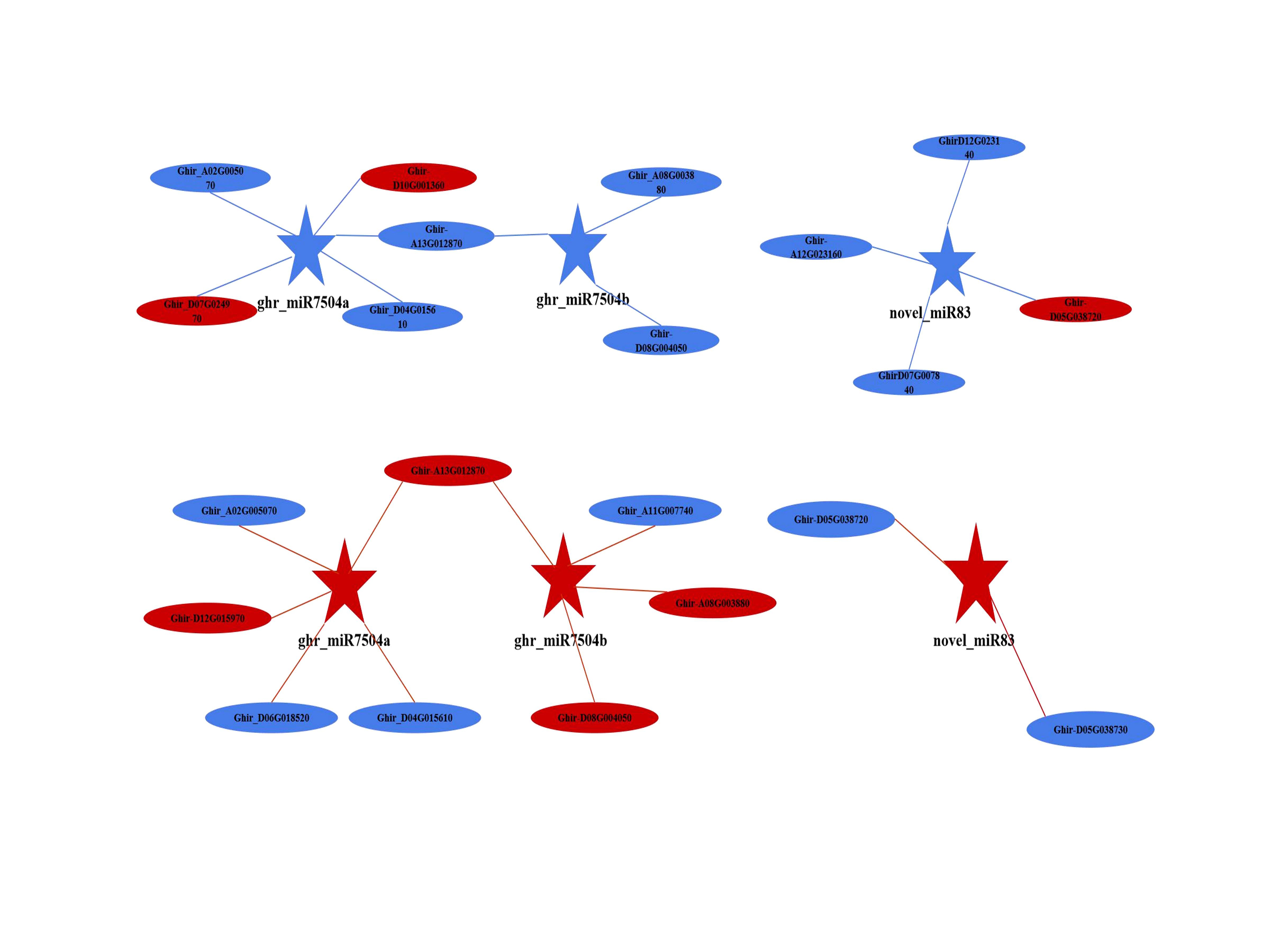

Supplement: Supplementary Figure 4 — Vis Network of the interaction. Positive and negative regulation relationships are expressed in red and blue, respectively. [file Image_4.JPEG]

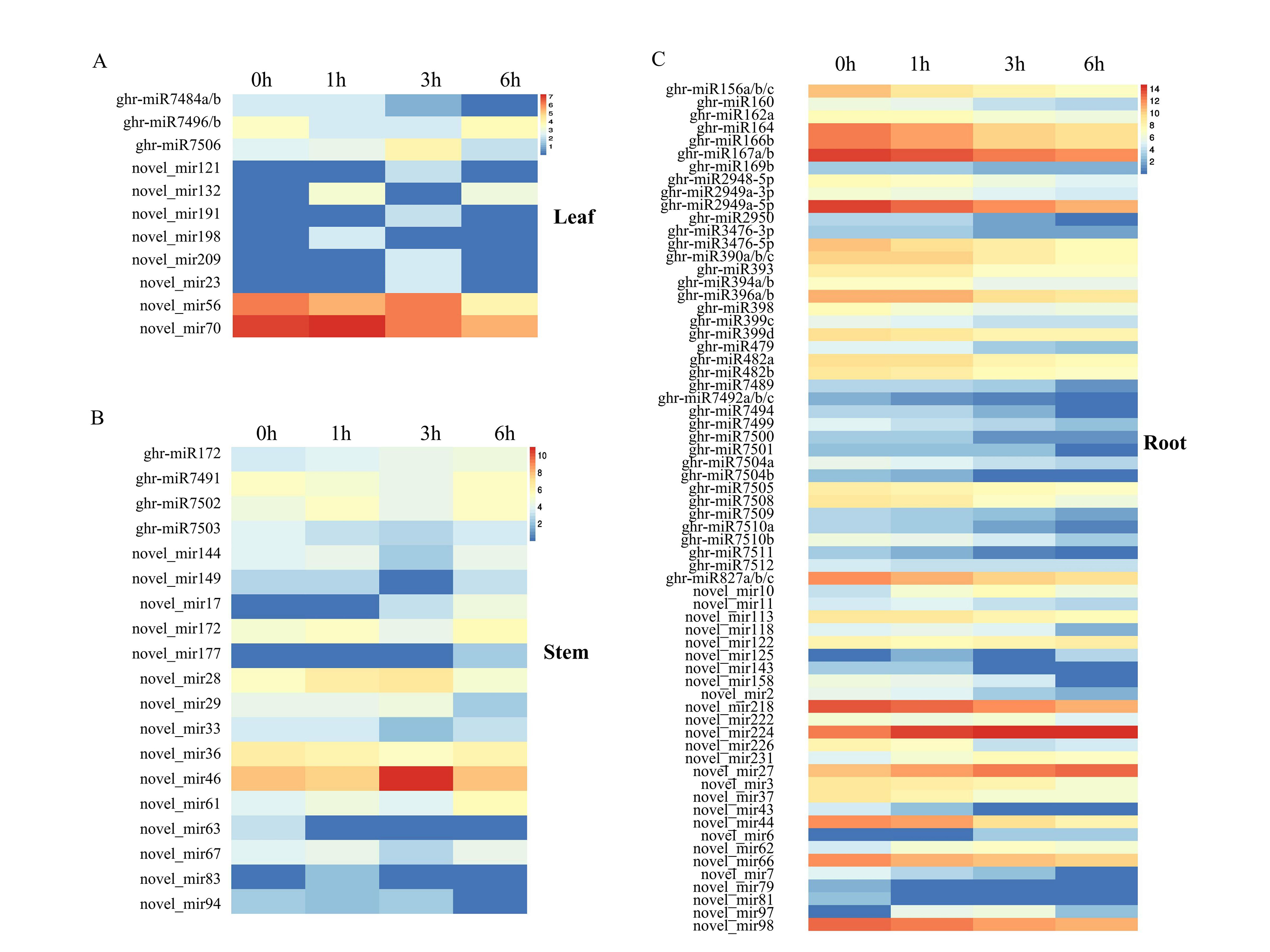

Supplement: Supplementary Figure 5 — Heat map of the expression patterns of specific differential expressed miRNAs in different tissues. (A) miRNA differentially expressed only in leaves. (B) miRNA differentially expressed only in stems. (C) miRNA differentially expressed only in roots. [file Image_5.JPEG]

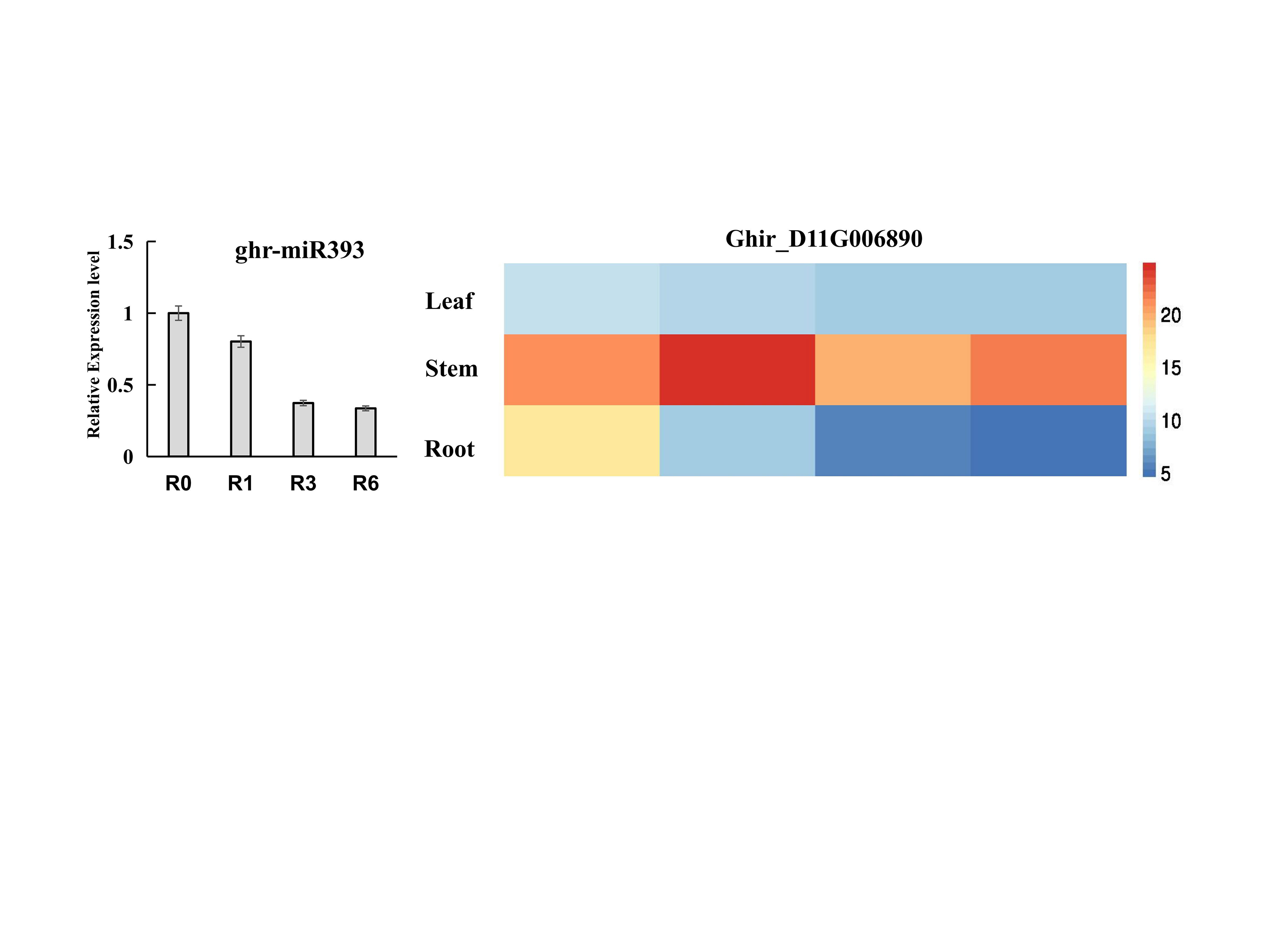

Supplement: Supplementary Figure 6 — The expression patterns of miR393 and its target. [file Image_6.JPEG]

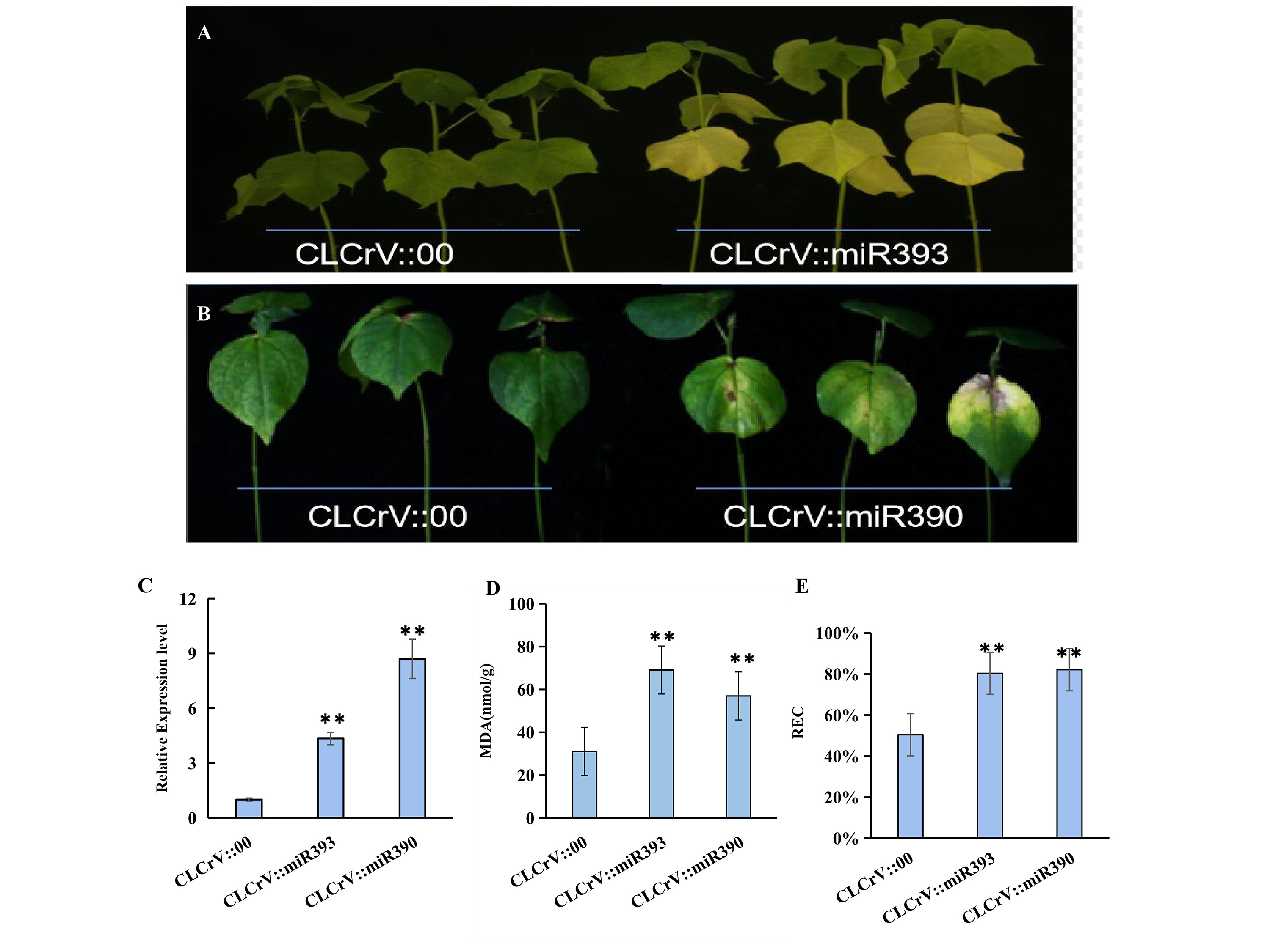

Supplement: Supplementary Figure 7 — (A,B) Phenotypes on cotton plant CLCrV:miR390, CLCrV:miR393 after NaCl treatment. (C) Relative expression of miRNA (miR390, miR393) in leaves of cotton plants. (D) Determination of malondialdehyde content in leaves of cotton. (E) Relative electric conductivity in leaves of cotton. [file Image_7.JPEG]

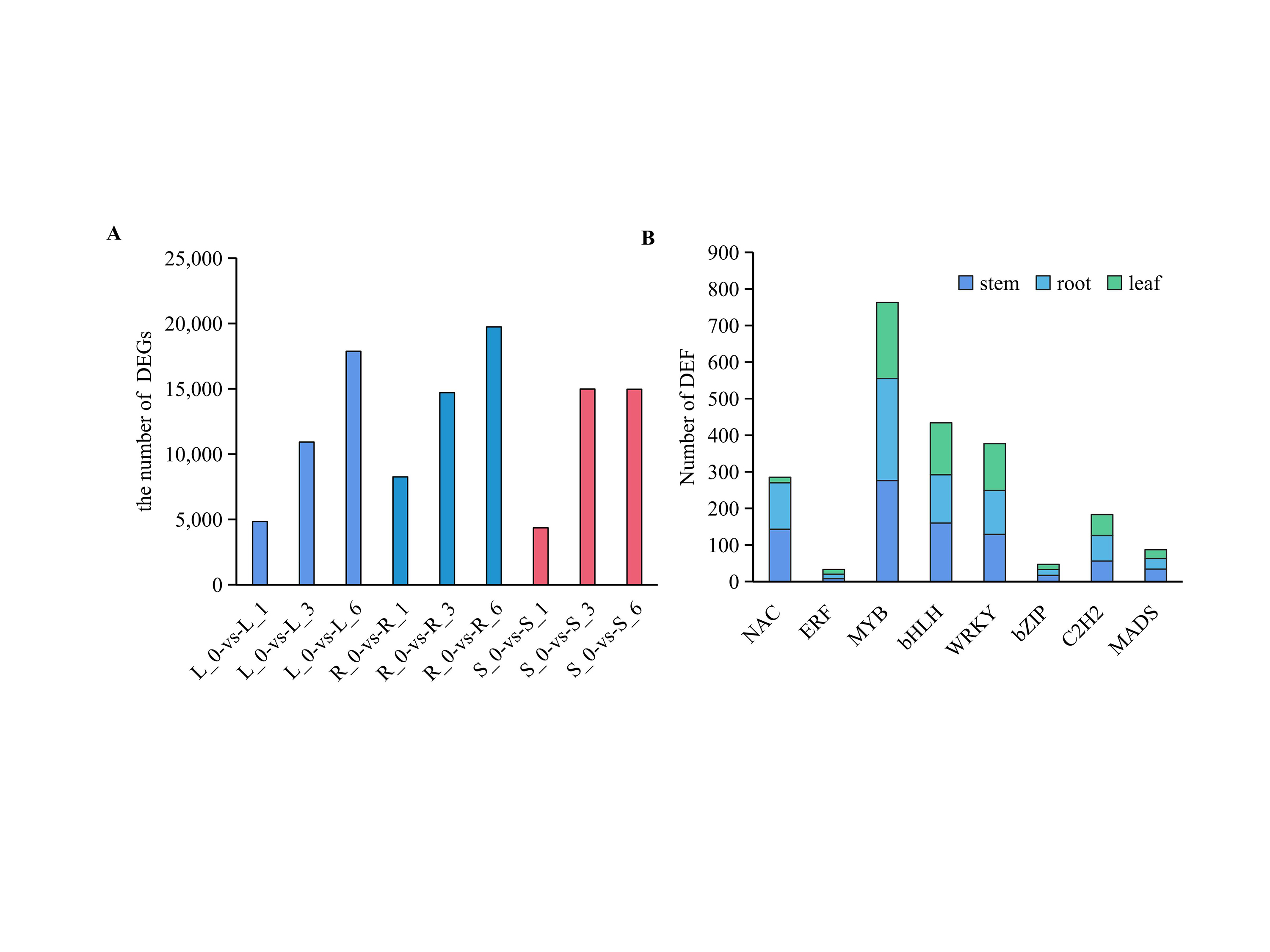

Supplement: Supplementary Figure 8 — (A) Number of differential expression genes in different organs. (B) Number of differentially expressed TF families in leaves, roots, stems. [file Image_8.JPEG]
